# The Effects of PLGA Nanoparticles Containing Different Growth Factors on Neural Stem Cell Differentiation and Their Transition Efficiency After Targeting With TRF

**DOI:** 10.1111/cns.70576

**Published:** 2025-09-04

**Authors:** Ayşegül Açıksarı, Yusufhan Yazır, Serap Mert, Zehra Seda Halbutoğulları, Sümeyye Narin, Gülçin Gacar

**Affiliations:** ^1^ Center for Stem Cell & Gene Therapies Research & Practice Kocaeli University Kocaeli Turkey; ^2^ Department of Stem Cell, Institute of Health Sciences Kocaeli University Kocaeli Turkey; ^3^ Sabanci University Nanotechnology Research and Application Center (SUNUM) Istanbul Turkey; ^4^ Department of Histology and Embryology, Faculty of Medicine Kocaeli University Kocaeli Turkey; ^5^ Department of Polymer Science and Technology Kocaeli University Kocaeli Turkey; ^6^ Department of Chemistry Faculty of Art and Science Kocaeli University Kocaeli Turkey; ^7^ Department of Medical Biology, Faculty of Medicine Kocaeli University Kocaeli Turkey

**Keywords:** neural stem cells, Parkinson's disease, PLGA nanoparticles, targeting

## Abstract

**Aims:**

Nanoparticle‐mediated drug delivery systems are being investigated for the controlled release of drugs to treat neurodegenerative diseases (ND). We aimed to investigate the effects of poly(lactic‐co‐glycolic acid) nanoparticles (PLGA‐NPs) containing different growth factors (GFs) on rat brain‐derived neural stem cells (NSCs) in vitro differentiation, providing insights that may contribute to future approaches for treating Parkinson's disease.

**Methods:**

Three different PLGA‐NPs loaded with Brain‐Derived Neurotrophic Factor (BDNF), Glial‐Derived Neurotrophic Factor (GDNF), and Transforming Growth Factor beta 3 (TGF‐β3) were developed and characterized in terms of size, zeta potential, encapsulation efficiency, and release profile. These NPs were used to differentiate NSCs into dopaminergic neurons in vitro. Additionally, the transition of transferrin (TRF)‐conjugated PLGA‐COOH‐NPs across an in vitro blood–brain barrier (BBB) model was investigated.

**Results:**

The average sizes of BDNF, GDNF, and TGF‐ß3 loaded PLGA‐NPs were measured to be 217.17 ± 1.37, 227.37 ± 5.39, and 220.57 ± 10.10 nm, respectively. Besides, SEM imaging revealed that the particles had a homogeneous size distribution and smooth surface morphology. Microtubule‐associated protein 2 (Map2) and tyrosine hydroxylase (TH), two dopaminergic neuronal markers, were found in cells with neuron‐like morphology that were produced through in vitro differentiation. The cellular uptake of PLGA‐NPs loaded with Coumarin‐6 was determined by using confocal imaging and flow cytometry. It was demonstrated that TRF‐conjugated NPs were specifically targeted and taken up into NSCs in the in vitro BBB model.

**Conclusion:**

It is concluded that BDNF‐PLGA‐NPs, GDNF‐PLGA‐NPs, and TGF‐ß3‐PLGA‐NPs are promising brain drug delivery carriers for NSC inducers, which could be useful in developing strategies for Parkinson's disease management, particularly when targeted with TRF.

## Graphical abstract

Demonstrating growth factor‐loaded PLGA‐NPs in vitro usage to differentiate dopaminergic neurons from NSCs for Parkinson's disease treatment and investigation targeting efficiency of TRF‐conjugated NPs in an in vitro BBB model.

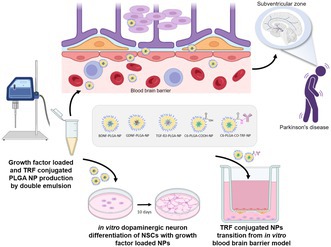

## 1 Introduction

Neurodegenerative diseases (NDs) are systemic diseases that occur following structural and functional neural loss [1]. Current treatment methods are mostly pharmacological, but they are not sufficient [2]. In particular, they are not aimed at solving the cause of the disease, but rather at suppressing its side effects or preventing its progression. Parkinson's disease is one of the most common NDs following the death of dopaminergic neurons in the substantia‐nigra region of the brain [3, 4, 5]. Stem cells have been widely used in studies in recent years to enable the generation of new neurons and thereby cure Parkinson's disease.

Stem cells are immature cells with the ability to self‐renew and divide without limit. Stem cells with different abilities can be obtained from different sources. Neural stem cells (NSCs) are promising cells that can form neural series and induce neurogenesis. They are promising for ND treatments [6, 7]. There are neurotrophic growth factors (GFs) and cytokines that enable NSCs to differentiate into new cell lines [8, 9]. GFs are important biomolecules because of their role in neuronal survival, differentiation, and maintenance of mature neurons. GFs have been investigated in many studies for use in the treatment of different diseases by loading them into polymeric nanoparticles (NPs) [6, 10].

Polymeric NPs have been researched for 20 years, especially for the treatment of NDs. The developed polymeric NPs aim to provide the required amount of drug release to the desired site, as well as to maintain drug efficacy and regeneration [11, 12]. Polymeric NPs with diameters between 1 and 1000 nm can be produced from natural polymers or synthetic polymers such as (poly(lactic‐co‐glycolic acid)) (PLGA) and polylactic acid (PLA) [11, 13]. These materials are biocompatible and biodegradable, and the monomers obtained after their degradation are known to be naturally eliminated from the body and non‐toxic [14]. Moreover, drug delivery systems are one of the current therapeutic approaches developed to maintain the efficacy of a therapeutic agent, control the amount of effect, or identify the target site. All of these characteristics allow NPs to be created to target a wide range of bodily locations. There are few treatment options, particularly because of limited access to the spinal cord and brain areas. Therefore, NP‐mediated transport mechanisms are very important to exceed the blood–brain barrier (BBB) [11].

The BBB is a critical barrier in the treatment of NDs. Overcoming the BBB, which limits the entry of active compounds or drugs into the brain, with a carrier system such as NP holds promise for the development of novel and customized therapies [13, 15]. NPs made of HPG (hyperbranched polyglycerol) and PLGA polymers were shown to be directed to brain microvascular endothelial cells after functionalization with OX26 (transferrin [TRF] antibody). The endomorphin‐loaded targeted NPs produced in the study were shown to provide analgesic effects in rats with chronic constriction injury [16]. Anti‐TRF (OX26) and anti‐amyloid ß (DE2B4) coated PLGA particles were developed for the treatment of Alzheimer's disease, and the migration of amyloid beta peptide was investigated in an in vitro BBB model [17]. The researchers discovered that the functionalized nanoparticles significantly enhanced the controlled uptake of the peptide into brain endothelial cells.

Studies have shown that biodegradable polymeric particles developed on the basis of nanotechnology trigger the stimulation and differentiation of stem cells by slow release of loaded proteins, thus bringing about regeneration in the microenvironment where the cells are located [18]. Dopamine‐carrying polymeric NPs have been used as an alternative treatment for Parkinson's disease [19]. In addition, GF‐loaded nanocarriers have been produced to obtain dopamine‐producing cells, and their effectiveness was investigated with in vivo and in vitro experiments in the literature [19, 20, 21, 22].

In this study, we attempted to establish a PLGA‐NP system that separately carries three different GFs and enables controlled release to NSCs to direct dopaminergic neuron differentiation in vitro. BDNF, GNDF, and TGF‐ß3 are preferred as GFs because they are effective in dopaminergic neuron differentiation, which is particularly lost in Parkinson's patients [4, 23]. Moreover, the in vitro BBB model was established using pericytes, Human Umbilical Vein Endothelial Cells (HUVECs), and NSCs, and then the transition of TRF conjugated and C6 labeled PLGA‐NPs, through this model was examined by flow cytometry and confocal microscopy. NSCs obtained from rat brain tissue were stimulated with three different GF‐loaded NPs in vitro for the first time, and the differentiation potential of the cells into dopaminergic neurons was examined by gene expression analysis and immunofluorescence staining for tyrosine hydroxylase (TH), beta III Tubulin (Tubb3) and microtubule‐associated protein 2 (Map2) to understand the molecular mechanism.

## 2 Materials and Methods

### 2.1 Preparation of PLGA‐NPs

**FIGURE 1:**
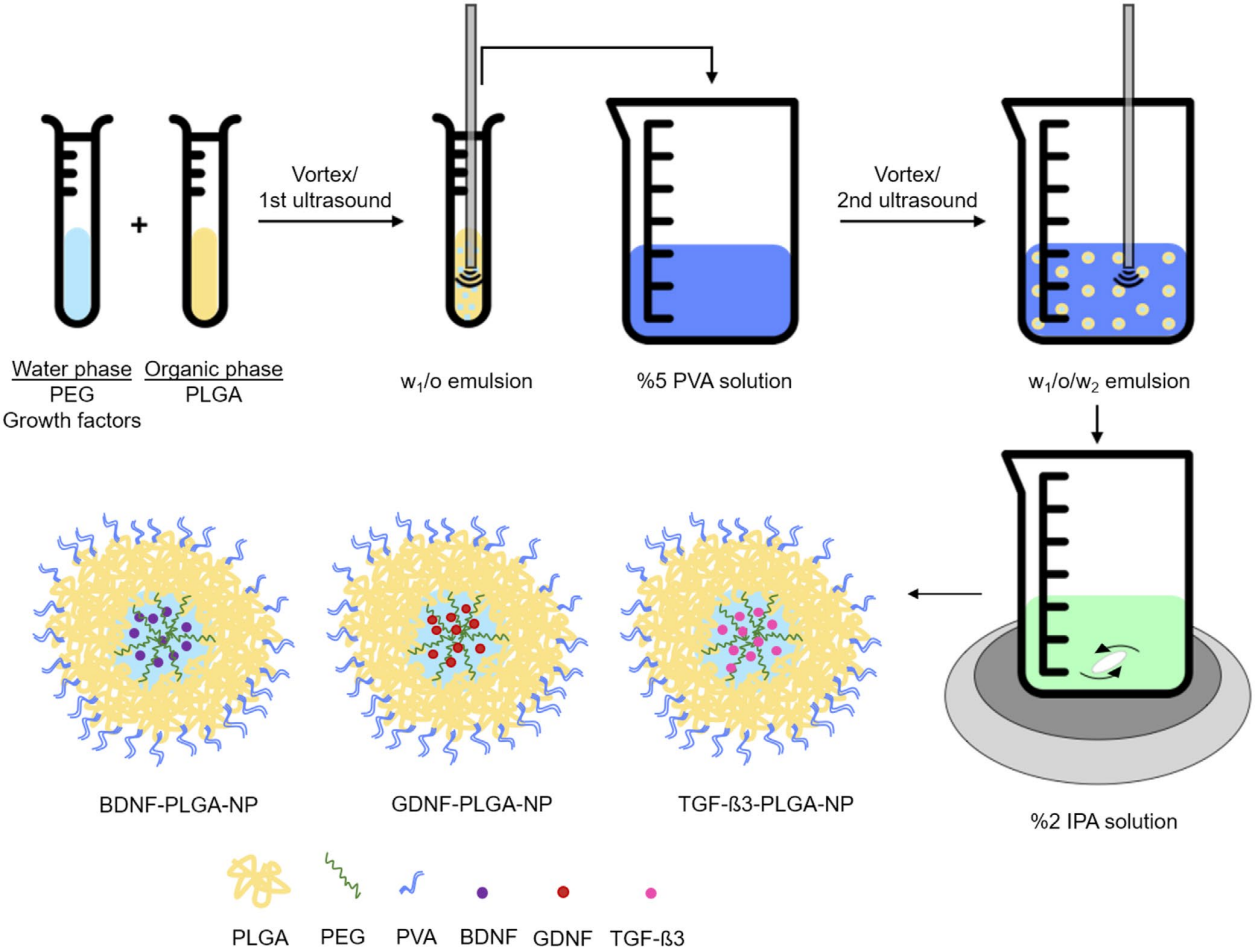
Experimental design for nanoparticle preparation method.

Three different NPs loaded with BDNF, GDNF, TGF‐ß3 were prepared separately by the double emulsion and solvent evaporation (w1/o/w2) method using poly(lactic acid‐co‐glycolic acid) (PURASORB PDLG 5010 PLGA 50/50, Corbion, Amsterdam, The Netherlands) and polyethylene glycol (PEG; average Mn 400, Sigma‐Aldrich, Steinheim, Germany) according to a modified protocol pioneered in the literature (Figure 1) [24]. The process involved adding GF droplets to PLGA in a mixture, resulting in a binary phase. The initial emulsion was formed by vortexing and sonication, entrapping GFs in PLGA spheres. The second emulsion was obtained by adding 5% polyvinyl alcohol and sonication. The two‐layer NP mixture was transferred to 2% IPA and stirred for 2 h. The NP suspension was ultracentrifuged at 12,000 rpm at +4 ^o^C and washed with dH_2_O. The obtained NPs were freeze‐dried in a lyophilizer (Labconco, FreeZone 2.5 Plus) for 24 h and stored at −20°C. In in vitro experiments, NPs were added to the medium, kept in a biosafety cabinet under UV‐C irradiation for 30 min for sterilization, and used directly after production without freeze‐drying [25, 26]. For cellular uptake studies, the fluorescent active agent coumarin‐6 (C6, 3‐(2‐benzothiazolyl)‐N, N‐diethylumbelli‐ferylamine, 3‐(2‐benzothiazolyl)‐7‐(diethylamino)coumarin; Ex/Em: 495/520 nm; Sigma–Aldrich, Steinheim, Germany)‐loaded PLGA‐NPs (C6‐PLGA‐NPs) were prepared with the same protocol by using 5% (w/v) C6 instead of GF [27]. Furthermore, the cytotoxic effects of the produced PLGA‐NPs on fibroblast growth were assessed using the WST‐1 assay.

### 2.2 Anti‐TRF Antibody Conjugation to PLGA‐NPs

**FIGURE 2:**
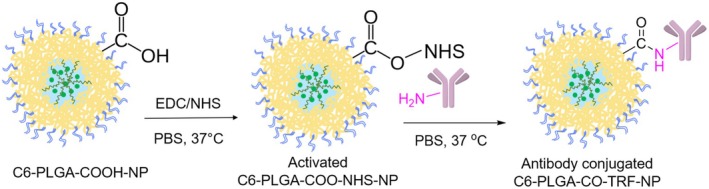
Conjugation of C6‐PLGA‐CO‐TRF‐NP with TRF antibody.

PLGA‐COOH polymer (acid‐terminated, lactide:glycolide 50:50, Sigma‐Aldrich, Steheim, Germany) was used to provide covalent bonding between Anti‐TRF (10 μg/mL) and the polymer by using EDC (1‐ethyl‐3‐[3‐dimethylaminopropyl]‐carbodiimide) and NHS (N‐hydroxysuccinimide) according to the literature (Figure 2) [16, 28, 29]. The micro‐BCA (bicinchoninic acid) protein assay (Boster Bio) was used to determine the conjugation efficiency. Then, the size and zeta potential of the obtained TRF‐conjugated NPs were measured.

### 2.3 Characterization of Nanoparticles

#### 2.3.1 Particle Size, Zeta Potential, and Electron Microscopy

The Malvern Zetasizer Nano ZS90 was used to assess the mean particle diameter, size distribution, and zeta potential of the produced NPs [24]. The morphology of the NPs was observed by scanning electron microscopy (SEM) to determine their size distribution and surface characteristics. For SEM imaging, freeze‐dried NPs were resuspended in distilled water. The samples were subsequently sputter‐coated with Au/Pd for 120 s at 40 mA current with a Cressington Sputter Coater 108. SEM micrographs were acquired with a scanning electron microscope (LeoSupra 35VP FE‐SEM, Zeiss) at 3 kV.

#### 2.3.2 The Release Profile of NPs and Encapsulation Efficiency

The GF‐loaded NPs (9 mg) were separately pre‐treated with 1 M NaOH overnight and neutralized with 1 M HCl, followed by centrifugation at 2000 rpm for 5 m. Empty NPs were subjected to the same treatment and used as a blank for calculation. Then micro‐BCA protein assay was used to determine the total amount of GF loaded into the NP. Furthermore, the total amounts of GF were checked using a 30‐day release experiment. Loaded and unloaded NPs (9 mg) were suspended in 0.5 mL PBS and incubated at 37°C and 1000 rpm with rotation to determine release profiles. The NPs were centrifuged at 11000 rpm for 10 m at 4°C at specific time intervals on days 1, 10, and 30, and the supernatants were collected. The amount of protein released into the supernatants was determined by micro BCA protein assay according to the manufacturer's recommendations [22, 30]. With this experiment, both the GF released from the NP over 30 days and the total amounts of GF loaded into the NP were determined.

### 2.4 NSCs Culture and Differentiation to Dopaminergic Neurons

NSCs derived from fetal rat brain tissue obtained from our previous study in our laboratory were used [31, 32]. These cells were obtained enzymatically from the lateral ventricle and stored at −152°C in cryopreservation medium containing 35% fetal bovine serum, 60% DMEM‐F12, and 5% DMSO [33]. After rapid thawing, cells were proliferated in culture, and characterization experiments were repeated for this study (Figure S5).

NSCs were grown in suspension culture flasks at 37°C in an incubator with 5% CO_2_ with neural supplement (StemPro; 1×), B27 supplement (1×, Gibco), FGFb (fibroblast GF basic; 20 ng/mL), EGF (epithelial GF; 20 ng/mL), heparin (0.02%), Glutamax (1×), and pen/strep (1%) [34]. The medium was renewed every 2 days, and cells were passaged when neurospheres exceeded 100 μm. For passaging, cells were collected in 15 mL Falcon tubes and centrifuged for 5 m at 90 g to form a pellet. The pellet was resuspended with 180 μL of Trypsin EDTA 0.25% (Gibco) and pipetted 10–15 times to form a single‐cell solution for counting on a Thoma slide. Upon withdrawal of bFGF and heparin from the culture medium, NSCs were transferred to adherent culture conditions to induce spontaneous differentiation, and the expression of various neural markers was assessed for characterization purposes.

NSCs were seeded in 6‐well (3 × 10^5^ cells/well) culture plates coated with Poly‐L‐Ornithine (20 μg/mL) and laminin (5 μg/mL) to differentiate dopaminergic neurons using GF‐loaded NPs (BDNF‐NP, GDNF‐NP, TGF‐ß3‐NP). FGFb and heparin were removed from the nutrient medium, and N2 supplement (1%), BDNF‐PLGA‐NP (0.750 mg/mL), GDNF‐PLGA‐NP (0.750 mg/mL), TGF‐ß3‐PLGA‐NPs (0.075 mg/mL), ascorbic acid (AA) (0.2 mM), and cAMP (0.5 mM) were added. Cells were grown in adherent culture for 10 days, and B‐27 supplement (1×) was added after day 6. In addition, to better observe the effects of NPs and recombinant protein versions on NSCs, free recombinant protein versions of BDNF (20 ng/mL), GDNF (20 ng/mL), and TGF‐ß3 (1 ng/mL) were used to differentiate NSCs according to the literature protocol [4, 23]. BDNF‐PLGA‐NP and GDNF‐PLGA‐NP were added into the culture medium at a concentration of 0.750 mg/ml, based on their release rates, to ensure a sustained daily release of approximately 20 ng/ml of each GF, maintaining the consistency with the chemical induction protocol. However, because the chemical induction experiment employed 1 ng/mL of TGF‐ß3, and TGF‐ß3‐PLGA‐NPs were diluted 10‐fold for the differentiation experiment. The same experiment was also performed with empty PLGA‐NP, which is equal the total amount of GF‐loaded PLGA‐NPs. Morphological changes in cells were observed under an inverted microscope (Olympus, IX71).

The differentiation experiments of NSCs into dopaminergic neurons included differentiation groups in which the three GFs were carried by the NP, as well as experimental groups in which the GF‐loaded NP was added in single and double combinations and comparatively examined (Table S1).

### 2.5 *In Vitro* BBB Model

An in vitro BBB model was established using NSCs, HUVECs, and pericytes. Pericytes were isolated from rat brain and cultured with endothelial cell growth medium (ECGM, Lonza). These cells were characterized by immunofluorescence staining (Figure S6). Membrane filters (Translucent High‐Density Polyethylene Terephthalate Membrane, BD Life Sciences) with a pore size of 3 μm and 12‐well plates were used. NSCs placed on the bottom of the culture plate below the barrier represented the brain region (basolateral region), whereas the top of the barrier was made to mimic blood (apical region) [35]. (Figure 4).

First, the basolateral side of the membrane filters was coated with poly‐L‐lysine (0.1%, Sigma Aldrich) for 1 h. The membranes were incubated upside down for 3 h and cultured with ECGM for 48 h to allow pericytes (2 × 10^4^/well) to seed onto the coated surface. HUVECs were cultured in DMEM‐F12 medium containing 10% FBS, 1% ITS (insulin, TRF, selenium; Gibco, Thermo Fisher Scientific), endothelial cell growth supplement (ECGM; 50 μg/mL, Sigma Aldrich), and pen/strep for 48 h to cover the surface and form a barrier‐like structure through tight junctions. After seeding NSCs (1 × 10^5^/well) in a separate 12‐well culture plate, the cells were incubated for 48 h; whereupon a co‐culture was initiated for 72 h to form the BBB microenvironment. Following the 5^th^ day, the apical region was treated with HUVEC medium and the basolateral region was treated with a 2:1 mixture of pericyte and NSC medium. C6‐PLGA‐NPs, C6‐PLGA‐COOH‐NPs, and C6‐PLGA‐CO‐TRF‐NPs were added on the 8^th^ day and incubated for 3 and 24 h to examine their uptake into NSCs via the in vitro BBB model. Uptake into NSCs was analyzed by flow cytometry (Software Kaluza Analysis 2.1, Beckman Coulter, Navios EX Flow Cytometer) and fluorescence microscopy (Leica, SP8 Confocal Laser Microscope).

### 2.6 Gene Expression Analysis of Cells

To analyze gene expression in differentiated cells, total RNA was isolated using the GeneJet RNA Isolation Kit (Thermo Fisher Scientific, USA) according to the manufacturer's instructions. Following single strand cDNA synthesis with the RevertAid First Strand cDNA Synthesis Kit (Thermo Fisher Scientific, USA), gene expression levels were determined on a Real‐Time PCR instrument (Lightcycler 480, Roche) using SYBR green master solution (RealQ Plus Master Mix Green, Ampliqon) and gene‐specific primers (Table S2). Data were processed using LC480 SW1.5 software. Expression levels of differentiated cells were compared with control group cells (NSCs).

### 2.7 Immunostaining of Cells and Imaging

Cells were rinsed with PBS and fixed in ice‐cold 4% PFA for 15 m. Samples were permeabilized with 0.025% Triton X‐100 (Merck) before being treated for 30 m in PBS containing 1.5% block serum (Santa Cruz Biotechnology, USA). Samples were treated with primary antibodies (Santa Cruz Biotechnology, USA) overnight at 4°C, followed by 2 h at room temperature. After three times 2 m PBS washes, cells were incubated with fluorescently labeled secondary antibody (Santa Cruz Biotechnology, USA) for 1 h at room temperature. Finally, cells were mounted with DAPI (4′,6‐diamidino‐2‐phenylindole, Santa Cruz Biotechnology, USA) and analyzed using a fluorescence microscope (Leica DMI 4000 Microsystem, Germany).

### 2.8 Demonstration of Cellular Uptake

To examine the direct passage of NPs into NSCs, cells were seeded at 1 × 10^5^/well in a 12‐well plate, and C6‐PLGA‐NPs were added to the culture medium (2.25 mg/mL) 24 h later. For microscopic examination, cells were incubated with C6‐PLGA‐NPs for 1 and 3 h. After 15 min room temperature fixation with 4% PFA, cells were covered with DAPI and observed using a confocal microscope (Leica, SP8 Confocal Laser Microscope) [36, 37]. NSCs were incubated with C6‐PLGA‐NPs for 24 h and dissociated with trypsin for examination by flow cytometer (Beckman Coulter, Navios EX Flow Cytometer, California) to measure fluorescence (495 nm excitation, 520 nm emission) [27]. Furthermore, TRF‐conjugated PLGA‐COOH‐NPs and unconjugated NPs were added to the in vitro BBB model to examine the efficiency of migration to NSCs after 3 and 24 h of incubation. C6‐free NPs were used as controls in all cellular uptake studies. Software Kaluza Analysis 2.1 program was used for cytometer analysis, and a total of at least 20,000 gated events were obtained for each sample.

### 2.9 Statistical Analysis

Each experiment was repeated at least three times, and the results were calculated taking into account the standard deviation. The data were analyzed using the unpaired Student's *t*‐test, the Newman–Keuls test, and analysis of variance for repeated measurements, where applicable. Differences between experimental and control groups were considered statistically significant at *p* < 0.05. (*p < 0.05, **p < 0.01, ***p < 0.001).

## 3 Results

### 3.1 PLGA‐NP Characterization

According to zeta sizer measurements, particle sizes of approximately 224.77 ± 0.93 nm for empty PLGA‐NP, 217.17 ± 1.37 nm for BDNF‐PLGA‐NP, 227.37 ± 5.39 nm for GDNF‐PLGA‐NP, 220.57 ± 10.10 nm for TGF‐ß3‐PLGA‐NP, and 225.10 ± 5.96 nm for C6‐PLGA‐NP were obtained by double emulsion experiments. PLGA‐COOH‐NPs were also found to have a size of around 220 nm (Table 1, Figures S1, S2).

**TABLE 1:** The mean particle diameter, size distribution, and zeta potentials.

Number	Nanoparticles	Size average (nm)	PDI	Potential (mV)
1	PLGA‐NP	224.77 ± 0.93	0.08 ± 0.03	−21.2 ± 1.44
2	BDNF‐PLGA‐NP	217.17 ± 1.37	0.05 ± 0.02	−23.1 ± 1.53
3	GDNF‐PLGA‐NP	227.37 ± 5.39	0.13 ± 0.04	−22.0 ± 0.17
4	TGF‐ß3‐PLGA‐NP	220.57 ± 10.10	0.14 ± 0.04	−22.3 ± 0.46
5	C6‐PLGA‐NP	225.10 ± 5.96	0.06 ± 0.01	−23.3 ± 0.62
6	PLGA‐COOH‐NP	220.93 ± 19.11	0.21 ± 0.06	−25.8 ± 1.23
7	C6‐PLGA‐COOH‐NP	210.20 ± 12.22	0.24 ± 0.06	−23.3 ± 0.62
8	PLGA‐CO‐TRF‐NP	283.27 ± 22.8	0.31 ± 0.01	−12.0 ± 0.64
9	C6‐PLGA‐CO‐TRF‐NP	275.67 ± 3.66	0.26 ± 0.02	−11.4 ± 0.80

**FIGURE 3:**
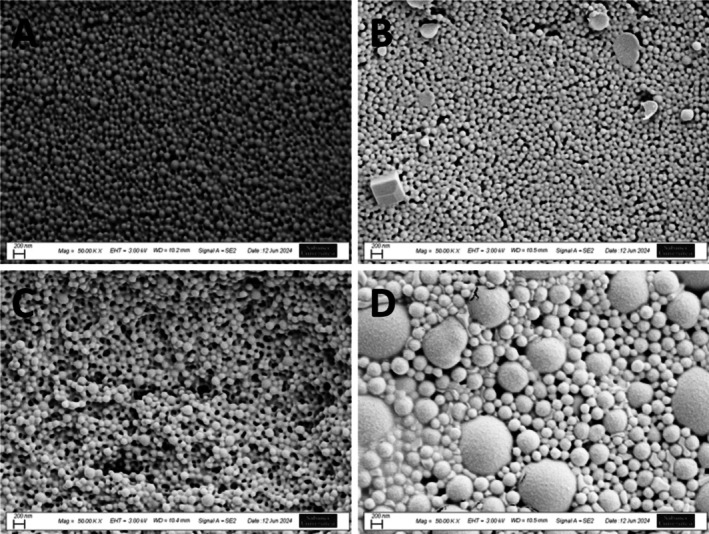
SEM images of PLGA‐NP (A), BDNF‐PLGA‐NP (B), GDNF‐PLGA‐NP (C), and TGF‐ß3‐PLGA‐NP after freeze‐drying (Scale bar: 200 nm, 50.00 K X magnification).

SEM images show that all particles have a smooth surface morphology after freeze‐drying (Figure 3). The figure shows that PLGA‐NPs (A) and BDNF‐PLGA‐NPs (B) have a homogeneous size distribution of 120 nm diameter. GDNF‐PLGA‐NPs (C) have the same size distribution but show some agglomeration after freeze‐drying. It was discovered that TGF‐ß3‐PLGA‐NPs contained some microparticles in the sample.

The zeta potential was similar for most formulations (Table 1; Numbers 1–7) and was around −22 mV. The size and zeta potentials of the NPs increased around 280 nm and −12 mV, respectively, after TRF conjugation (Table 1; Numbers 8, 9). The increase in zeta potential indicates the conjugation of antibodies on the NPs surface, as reported in the literature [29]. TRF binding efficiency of PLGA‐COOH‐NP was found to be 11% (1 μg TRF/3 mg NP) by calculating the non‐conjugated TRF amount in the supernatant by micro BCA assay (indirect method).

Table 2 shows that BDNF, GDNF, and TGF‐ß3 released from PLGA‐NPs exhibited varying release profiles, with initial release rates of 39.7%, 41.5%, and 25.2% of the total loaded protein within the first 24 h, respectively. According to the literature approach [22], since almost all of the protein in PLGA‐NPs is released at the end of 30 days, the protein encapsulation efficiency calculated on the basis of the amount of protein released in 30 days was 70% for BDNF‐PLGA‐NP, 68% for GDNF‐PLGA‐NP, and 93% for TGF‐ß3‐PLGA‐NP (Figure S3 and Table 2).

**TABLE 2:** *In Vitro* release profiles of GF‐loaded NPs at 37°C in PBS (pH 7.4).

Number	Nanoparticles	1^st^ Day Released GF amount (ng)	10 days released GF amount (ng)	30 days released GF amount (ng)	1^st^ day released GF (%)	GF encapsulation efficiency (%)^a^
2	BDNF‐PLGA‐NP	277.8	544.4	700.0	39.7	70
3	GDNF‐PLGA‐NP	283.3	344.4	683.3	41.5	68
4	TGF‐ß3‐PLGA‐NP	233.3	412.5	925.0	25.2	93

*^a^:* $GF\;encapsulation\;efficiency\;(\%)=\frac{30\;days\;released\;GF\;amount}{Amount\;of\;GF\;initially\;used}\times 100$.

### 3.2 Cellular Uptake of TRF Conjugated PLGA‐COOH‐NPs and in vitro BBB Model

**FIGURE 4:**
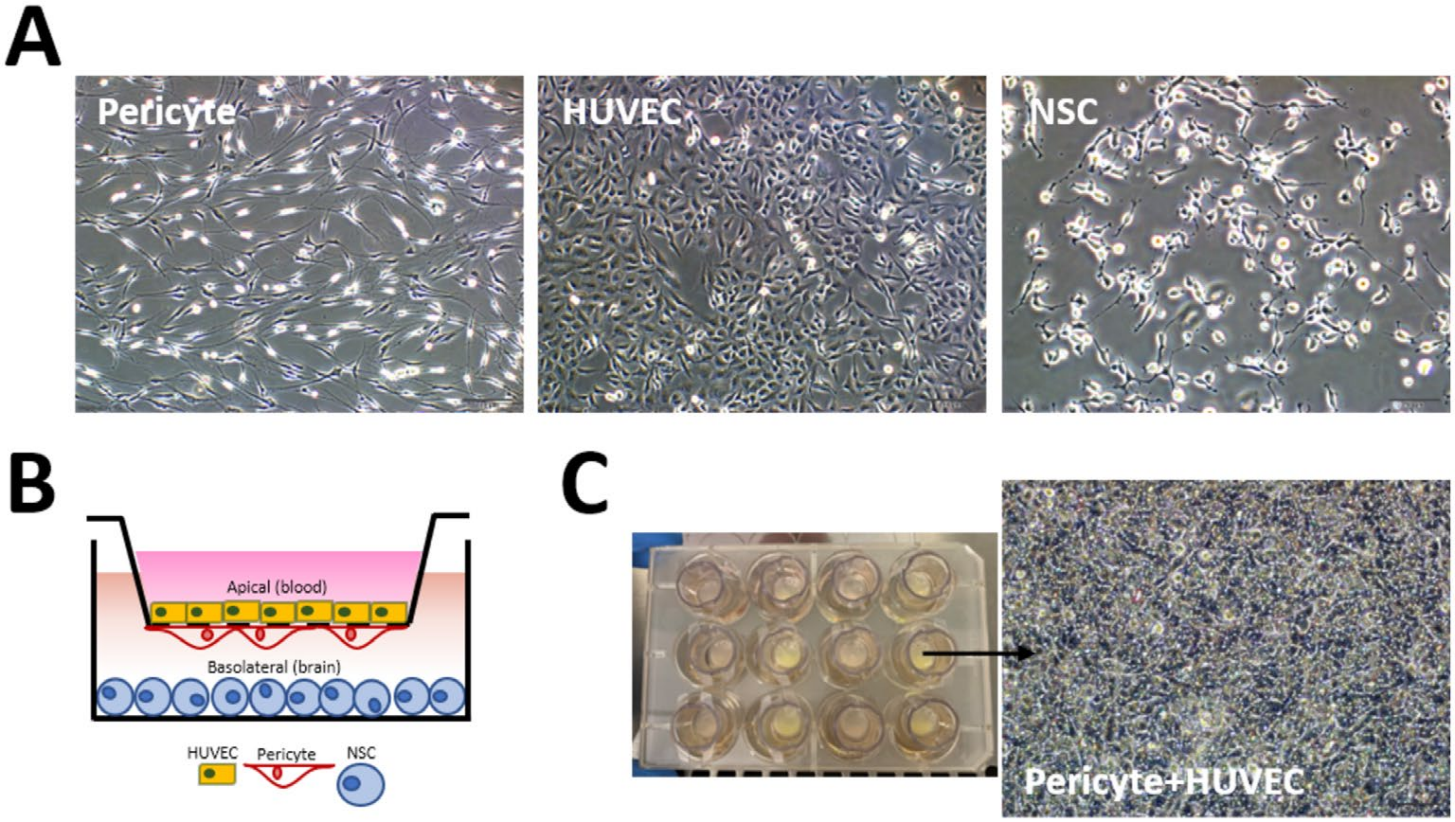
(A) Cellular images of pericytes, HUVECs, and NSCs used to create the in vitro BBB model. (B) Schematic representation of the in vitro BBB model. (C) Image of cells on the insert membrane, representing the BBB.

The in vitro BBB model cell structures were investigated with an inverted microscope (Figure 4). Pericytes have thin extensions, and the cobblestone‐like structure of HUVECs with large dark nuclei and NSCs as adherent cells were observed under an inverted microscope. These cells were employed to exhibit an in vitro BBB model, as illustrated in Figure 4B.

**FIGURE 5:**
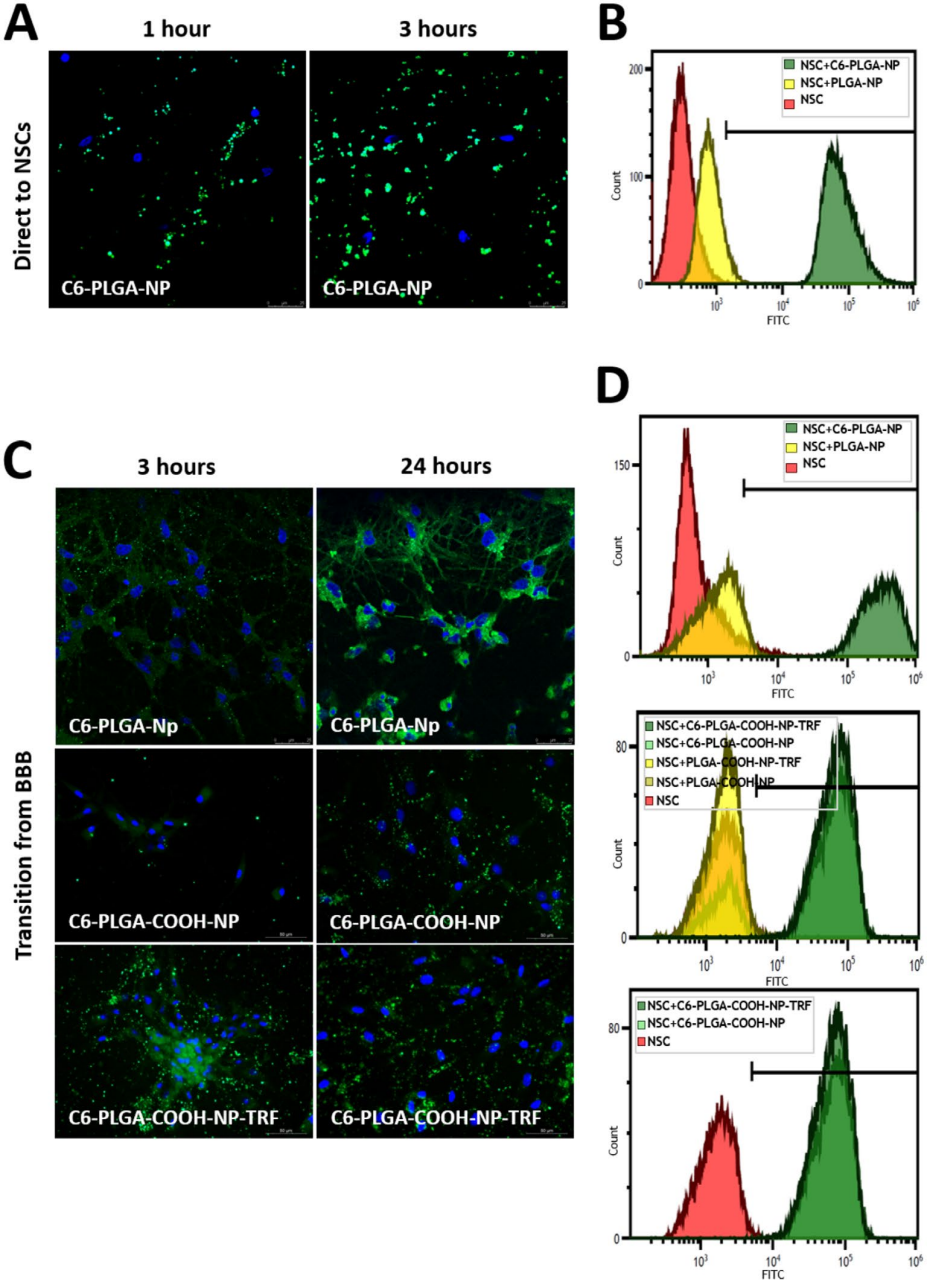
Imaging of the cellular uptake of NPs by NSCs directly (A, B) and through (C, D) an in vitro BBB model. Cell nuclei were stained blue with DAPI. The FITC channel was used to investigate C6‐loaded NPs (Scale bar; 25 and 50 μm).

It has been shown both by flow cytometry and confocal microscopy that the obtained NPs can be taken up into cells with their properties such as size and potential. Thus, it is thought that they can affect the differentiation of NSCs in vitro. The cellular uptake of the C6‐PLGA‐NPs into the NSCs was determined by fluorescence microscopy (Figure 5A) and flow cytometry (Figure 5B). Figure 5A shows that C6‐PLGA‐NPs could be internalized into the cells by fluorescence microscopy after 1 and 3 h of incubation. It was discovered that NP uptake increased as the incubation time increased, and more C6‐PLGA‐NPs were found in NSCs.

Similarly, when the three cell groups were compared by flow cytometry after 24 h of incubation, NSCs incubated with C6‐PLGA‐NP (green peak), the only group carrying a fluorescent marker, were found to be irradiated in the FITC channel. The control group (NSCs; red peak) and NSCs incubated with PLGA‐NPs (yellow peak) were observed in the negative region of the graph (Figure 5B).

In Figure 5C, it was observed by microscopy that NPs conjugated with the TRF antibody had a higher transit effect from the in vitro BBB model compared to the unconjugated ones, especially at 3 h of incubation. After 24 h, NPs carrying similar amounts of C6 (green) as TRF‐conjugated particles were detected in NSCs by flow cytometry (Figure 5D). The percentage results of uptaken NPs from the flow cytometry analysis are displayed in Table S3.

### 3.3 Differentiation of NSCs With NPs

**FIGURE 6:**
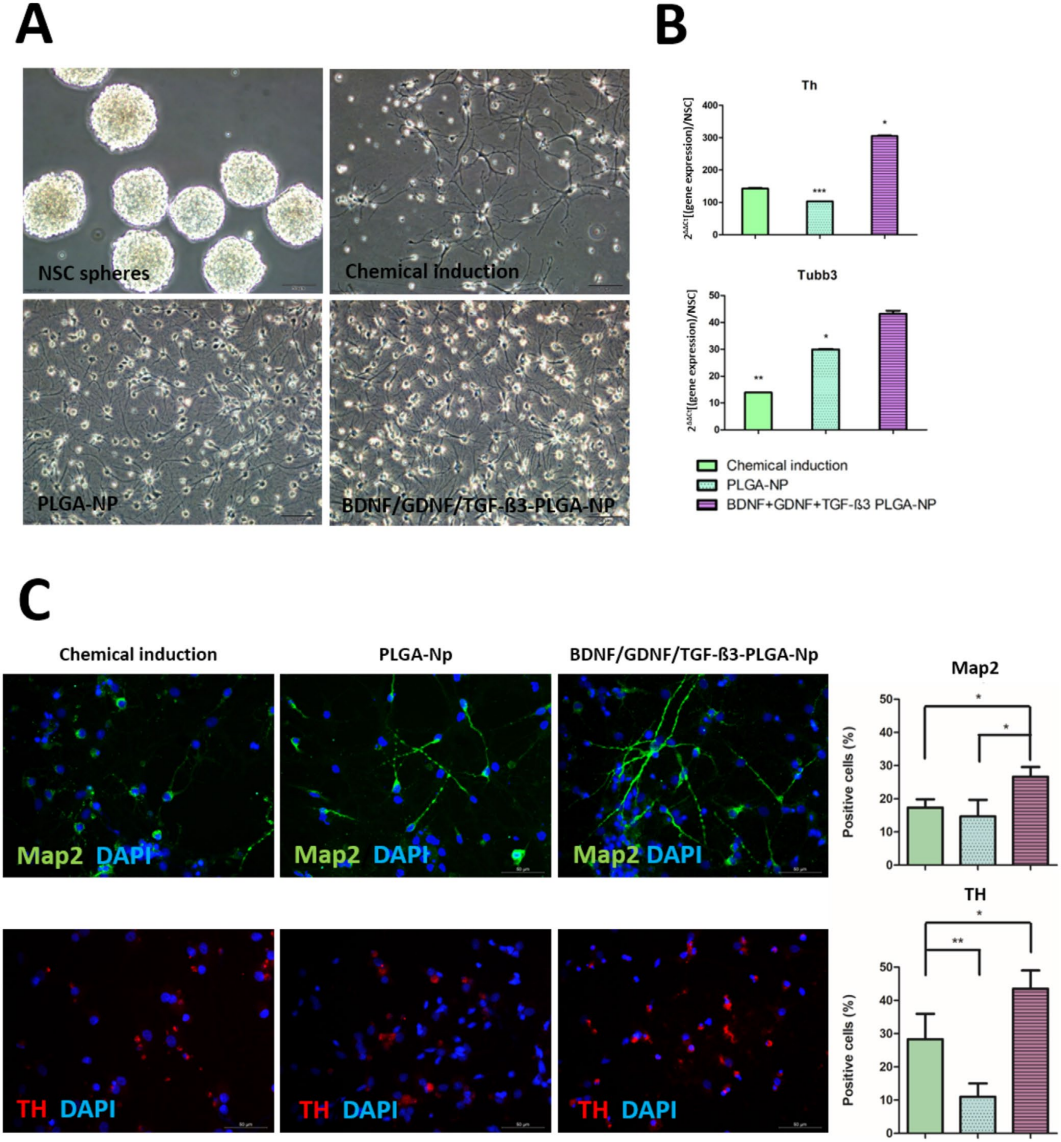
Cellular examinations after differentiation with GF‐loaded NPs. (A) Neurospheres (Control group) and imaging the cellular morphology of NSCs after differentiations at day 10 with an invert microscope. (B) Th and Tubb3 gene expression analysis of differentiated cells (*p < 0.05, **p < 0.01 as compared with NSC spheres). (C) Images of chemical induction group, PLGA‐NP differentiation group, and BDNF‐PLGA‐NP/GDNF‐PLGA‐NP/TGF‐ß3‐PLGA‐NP differentiation group after differentiation. Cell nuclei are shown in blue with DAPI, in green (FITC) for Map2, and in red (TR) for TH. (Scale bars: 50 μm) The percentages were calculated on the basis of the number of positively stained cells counted from the confocal images (*p < 0.05, *p* < 0.01 compared to the chemical induction group).

After the dopaminergic neuron differentiation consisting of the chemical induction group in which GFs were directly used, empty NPs and GF‐loaded NPs, the cells were observed under an inverted microscope (Figure 6A). During the experiment, the medium was not refreshed during the 10‐day period, and the NP was expected to be more effective than the chemical induction group by slow release. It was observed that cells with neuron‐like morphology were formed in all groups, and intercellular communication increased through cell extensions. It was noticed that PLGA‐NPs were effective on the differentiation of NSCs because of their biomechanical properties [6].

Chemical induction and PLGA‐NPs increased Th expression 142‐fold and 103‐fold in cells, respectively. However, the largest significant effect was seen in the BDNF‐PLGA‐NP/GDNF‐PLGA‐NP/TGF‐ß3‐PLGA‐NP differentiation group with 305‐fold relative to NSCs. Furthermore, Tubb3 expression increased 14‐, 30‐, and 43‐fold in the chemical induction, PLGA‐NP differentiation, and BDNF‐PLGA‐NP/GDNF‐PLGA‐NP/TGF‐ß3‐PLGA‐NP differentiation groups, respectively (Figure 6B).

Upon examination of Map2 staining, positive results were noted clustered around the cell nuclei in the chemical induction group, with 17.% ±2.51 of the cells exhibiting Map2 positivity. In contrast, more intercellular extensions were observed in the PLGA‐NP‐treated groups, with 15% ±5.03 of the cells expressing Map2 positivity. In the other differentiation group with GF‐carrying NPs, the proportion of cells exhibiting Map2 protein was elevated (27% ±2.88), and these cells displayed Map2 in clusters aggregated within their extensions. Statistical analysis verified that the proportion of Map2‐positive cells in this group was markedly elevated in comparison to the chemical induction group (*p < 0.05) (Figure 6C).

Positive staining surrounding cell nuclei was noted in all groups; however, akin to Map2 protein, TH protein did not extend into cellular extensions (Figure 6C). The chemical induction group had 28% ±7.63 TH‐positive cells, 11% ±4.00 PLGA‐NPs, and 43.5% ±5.50 GF‐loaded NPs. Notably, the group treated with three different GF‐loaded NPs demonstrated a significantly higher percentage of TH‐positive cells than the chemical induction group (**p < 0.01) (Figure 6C).

As seen in the images, empty NPs influenced NSC differentiation since NPs are known to activate stem cell signaling pathways (Figure 6A). According to the detailed results of Th and Tubb3 gene expression analysis, as well as the percentage of Map2‐positive cells, it was observed that this differentiation effect was greater in the group induced by three different GF‐loaded NPs (Figure 6B).

## 4 Discussion

NPs are increasingly recognized for their transformative potential in neurotherapeutics, particularly for overcoming the BBB, a major obstacle in treating neurological disorders. Recent studies have explored diverse NPs, including lipid‐based and polymeric nanoparticles, to enhance drug delivery to the brain. Surface modifications with targeting ligands and multifunctional nanoparticles for theragnostic purposes have further advanced the field [38]. In this study, PLGA‐NPs carrying BDNF, GDNF, and TGF‐ß3 were produced to enable sustained GF release, and thier in vitro cellular interations with NSCs from fetal rat brain tissue were investigated.

The primary source of BDNF and GDNF is astrocytes, which are known to fulfill a variety of tasks such as protecting the extracellular environment and stabilizing the communicatin between neurons and glial cells. The absence of GFs leads to neuronal loss and the progression of ND, highlighting their crucial role in preserving and protecting mature and injured neurons, making them a promising avenue for ND therapy [22]. BDNF has been linked to improved cognition, learning, memory formation, and modulation of synaptic plasticity in animal models, and a decrease in BDNF levels is observed in NDs such as Parkinson's. GDNF also prevents neuronal damage in response to ischemia and pain. It has been suggested that GDNF is vital for dopaminergic neurons and is a promising candidate for the treatment of Parkinson's disease [20]. Promoting BDNF and GDNF release is a promising therapeutic strategy for the treatment of neurological disorders [39, 40]. TGF‐ß3 is an important mediator in the induction and maintenance of midbrain dopaminergic neurons [41]. These factors, BDNF, GDNF, and TGF‐ß3, were chosen to load into NPs because of their crucial importance and documented effectiveness in encouraging in vitro differentiation processes to enable precursor cells to mature into dopaminergic neuron‐like cells [4, 23].

Double emulsion (w_1_/o/w_2_) method was preferred in our study to load hydrophilic proteins such as GFs [42], although there are many different NP production methods in the literature. It was shown that the NPs produced had similar size and potential values to those in the literature [24] and had no toxic effect on fibroblast cells (Figure S4).

The GF encapsulation efficiencies of BDNF, GDNF, and TGF‐β3 loaded nanoparticles were 70%, 68%, and 93%, respectively, which were higher than the literature [24]. This result was interpreted as indicating that the small amount of GF used at the start may have a positive effect. The GF release profiles of the produced nanoparticles demonstrated an initial burst on the first day, consistent with the literature, and the content was completely released at the end of 30 days [22, 24].

The TRF antibody conjugation yield was measured as 11%. According to the literature, antibody conjugation yield in NPs produced using PLGA‐PEG‐COOH copolymers ranges between 27% and 40% [28, 37]. Because the PLGA‐COOH polymer used in our study only contains –COOH at the end groups, it is hypothesized that the number of PLGA‐COOH polymer chains in the nanoparticle influences conjugation efficiency. Furthermore, the slower conjugation reaction of the hydrophobic PLGA‐COOH polymer in aqueous media compared to the more hydrophilic PLGA‐PEG‐COOH copolymer may be a limiting factor for efficiency. Therefore, the obtained 11% yield is considered an expected result.

According to the literature, in vitro BBB models created with brain‐derived microvascular endothelial cells produce more accurate results when evaluating NP passage. However, HUVECs are also used for this model [43]. Although this is one of the study's limitations, barrier formation was observed in the model created using HUVECs, pericytes, and NSCs. The passage differences between TRF‐conjugated nanoparticles demonstrated the effectiveness of the barrier and the faster passage of targeted particles.

Following differentiation, it was discovered that Th and Tubb3 expression in cells induced with GF‐loaded PLGA‐NPs increased roughly 300‐ and 40‐fold, respectively. Remarkably, previous studies have found that GFs can improve differentiation efficiency, with Th expression increasing 10‐ to 40‐fold under ideal conditions [44]. These findings highlight the efficacy of PLGA‐based delivery methods in promoting neuronal differentiation and serve as a foundation for building more efficient models for ND treatments.

NSCs are being investigated for the treatment of many neurological diseases because of their capacity to differentiate into different neuronal types and stimulate neuronal regeneration [45, 46, 47]. Parkinson's disease has recently been tried to be treated with NSCs. The idea of regenerating and replacing missing dopaminergic neurons from NSCs in the brain is being explored to provide a definitive treatment [48, 49]. In particular, nano‐sized carriers produced from biocompatible and biodegradable polymeric materials such as PLGA, which can cross the BBB and deliver active substances to the brain, show great promise [50]. Therefore, the goal of this study is to create a novel combination of three different GF‐loaded PLGA‐NPs and investigate their effects on the in vitro differentiation of NSCs, with potential implications for ND research.

Parkinson's rat models have been tried to be treated with NPs that carry dopamine and release it into the brain microenvironment [21]. Although improvement in behavioral disorders is observed, replacing the missing protein does not provide a definitive solution for this disease. The ability of NPs to reach and deliver their contents to NSCs in vivo without any surgical intervention, or the indirect effect of their contents being released in that microenvironment, drives differentiation and increases the number of dopaminergic neurons and regeneration. This approach holds promise as a Parkinson's disease management strategy [18, 51].

This study's ultimate objective is to evaluate these nanoparticles in vivo. Although there are in vivo studies investigating the effects of GF‐loaded NPs in the literature, there are very few studies that can be specifically directed to NSCs. The first study examining the effects of retinoic acid‐loaded PEI‐NPs on the differentiation of stem cells in vitro and in vivo was performed by Santos, et al. and Maia et al. [52, 53]. These studies proved that particles can be directed to NSCs and regeneration can be achieved by stimulating these cells. However, it provides a limited method because it uses NPs carrying a single agent. Many studies show that NSCs will form a somatic cell with a very important function, not with the help of a single agent, but with the use of different agents that will trigger many pathways [54]. It is hoped that assessing the in vivo efficacy of the created nanoparticles, which carry three separate GFs, would result in potential treatment approaches in the future.

Although our findings shed light on the potential of GF‐loaded NPs for NSC differentiation, some limitations of this study should be noted. First, all experiments were carried out in vitro with rat brain‐derived NSCs, which may not fully mimic the complexity of the in vivo environment in the human brain. Second, although the nanoparticle concentration was optimized for cytotoxicity in vitro and GF release, additional research is needed to validate their efficacy and safety in in vivo models. Third, the differentiation efficiency and long‐term effects of GF‐loaded NPs on NSCs were not studied after the initial observation period. Finally, this study did not assess NSC survival or proliferation under pathological conditions such as oxidative stress or exposure to inflammatory cytokines, which are critical in NDs. Future research will be required to address these limitations and further investigate the therapeutic potential of this nanoparticle‐based delivery system.

## 5 Conclusion

This study produced PLGA‐NPs with desired properties, carrying BDNF, GDNF, and TGF‐ß3 proteins and providing slow protein release. These NPs were used to differentiate NSCs and were evaluated in an in vitro BBB model. The Th gene expression levels of the cells increased 300‐fold when differentiated with GFs loaded PLGA‐NPs and were higher than those differentiated by chemical induction. The findings suggest that dopaminergic neuron‐like cells can be obtained using GF loaded PLGA‐NPs, cell differentiation can be achieved through controlled release, and these NPs can be used to regenerate degenerative brain tissue. Future research should evaluate these NPs in animal models of Parkinson's disease and investigate their therapeutic effects. This would allow proteins to be delivered more efficiently and protected from external factors while active, without the need for surgical intervention.

## Author Contributions

Y.Y. provided support in conceptualization, methodology, fund acquisition, project management, and auditing. A.A. and Y.Y. collaborated to develop the design of the study. A.A. and S.M. contributed to the manufacturing, characterization, and analysis of nanoparticle results. They also contributed to drafting the text and preparing the figures. A.A. and Z.S.H. cooperated to culture neural stem cells, differentiate them, and assess the results. G.G. helped with the collection and analysis of flow cytometer data. S.N. carried out the SEM imaging and measured the data.

## Ethics Statement

All animal experiments and procedures were approved by the Kocaeli University Animal Experiments Local Ethics Committee (KOÜ HADYEK 1/4‐2019).

## Conflicts of Interest

The authors declare no conflicts of interest.

## Supporting information


**Table S1:** All differentiation experiment groups.
**Table S2:** Primer sequences used in RT‐PCR.
**Table S3:** NP uptake percentage findings from flow cytometry.
**Figure S1:** Size distribution graphics of NPs (Malvern Zeta Sizer); PLGA‐NP (A), BDNF‐PLGA‐NP (B), GDNF‐PLGA‐NP (C), TGF‐ß3‐PLGA‐NP (D), C6‐PLGA‐NP (E), PLGA‐COOH‐NP (F), C6‐PLGA‐COOH‐NP (G), PLGA‐COOH‐NP‐TRF (H), and C6‐PLGA‐COOH‐NP‐TRF (I).
**Figure S2:** Zeta potential graphics of NPs (Malvern Zeta Sizer); PLGA‐NP (A), BDNF‐PLGA‐NP (B), GDNF‐PLGA‐NP (C), TGF‐ß3‐PLGA‐NP (D), C6‐PLGA‐NP (E), PLGA‐COOH‐NP (F), PLGA‐COOH‐NP‐TRF (G), and C6‐PLGA‐COOH‐NP‐TRF (H).
**Figure S3:** In vitro cumulative release profiles of growth factor‐loaded Np's at 37°C in PBS (pH 7.4). BDNF‐PLGA‐NPs (blue), GDNF‐PLGA‐NPs (red), and TGF‐ß3‐PLGA‐NPs (gray).
**Figure S4:** When NPs were applied at various dosages to fibroblast cells and compared to control group cells, it was shown that NPs had no statistically significant effect on cell proliferation. As a consequence, it was shown that NPs did not affect cell proliferation and had no toxic impact. The proliferative or cytotoxic impact of the NPs on fibroblast cells was determined by WST‐1 analysis.
**Figure S5:** Characterization of rat brain tissue‐derived NSCs at passage 3. Cell nuclei are labeled with DAPI (blue), GFAP, nestin, NG2, and S100 markers FITC (green) (Scale bar; 50 μm) (A). After 1 week of adherent culture, Nestin, Th, Tubb3, and Gfap gene expression levels of spontaneously differentiated NSCs were evaluated by RT‐PCR (B).
**Figure S6:** Characterization of rat brain tissue‐derived pericytes at passage 3. Cell nuclei are labeled with DAPI (blue), positive for PDGFRβ (A) and NG2 (B, C) markers FITC (green), and α‐SMA (A, B) marker TR (red). Negative for GFAP TR (red) marker (Scale bar; 50 μm).

## Data Availability

The data that support the findings of this study are available from the corresponding author upon reasonable request.
