# Supplementary figures and images for: The Effects of PLGA Nanoparticles Containing Different Growth Factors on Neural Stem Cell Differentiation and Their Transition Efficiency After Targeting With TRF
Source: CNS Neurosci Ther. 2025 Sep 4;31(9):e70576. doi: 10.1111/cns.70576 (PMC12409068; doi:10.1111/cns.70576)

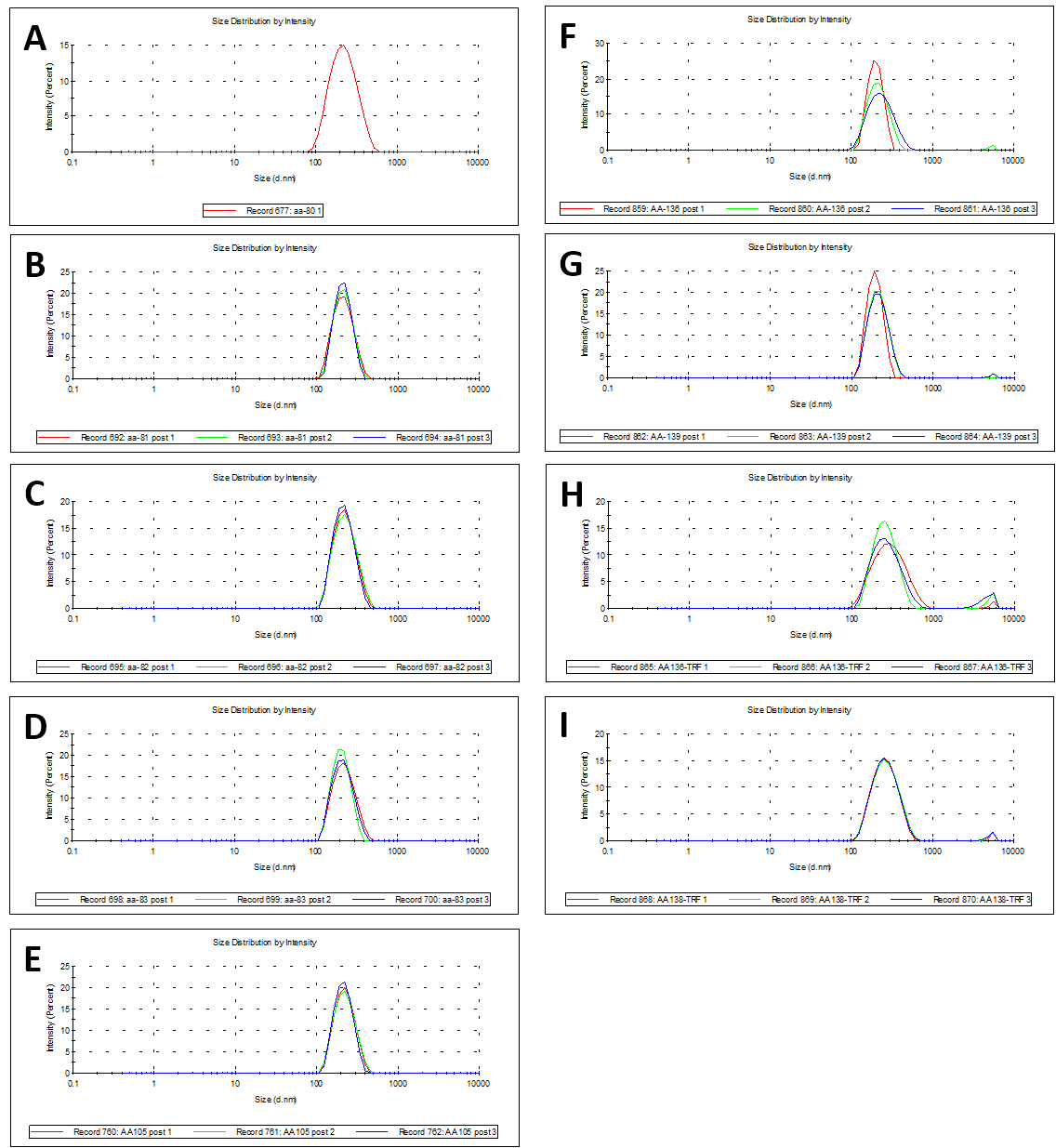

Supplement: Supplementary file 1 — Table S1: All differentiation experiment groups. Table S2: Primer sequences used in RT‐PCR. Table S3: NP uptake percentage findings from flow cytometry. Figure S1: Size distribution graphics of NPs (Malvern Zeta Sizer); PLGA‐NP (A), BDNF‐PLGA‐NP (B), GDNF‐PLGA‐NP (C), TGF‐ß3‐PLGA‐NP (D), C6‐PLGA‐NP (E), PLGA‐COOH‐NP (F), C6‐PLGA‐COOH‐NP (G), PLGA‐COOH‐NP‐TRF (H), and C6‐PLGA‐COOH‐NP‐TRF (I). Figure S2: Zeta potential graphics of NPs (Malvern Zeta Sizer); PLGA‐NP (A), BDNF‐PLGA‐NP (B), GDNF‐PLGA‐NP (C), TGF‐ß3‐PLGA‐NP (D), C6‐PLGA‐NP (E), PLGA‐COOH‐NP (F), PLGA‐COOH‐NP‐TRF (G), and C6‐PLGA‐COOH‐NP‐TRF (H). Figure S3: In vitro cumulative release profiles of growth factor‐loaded Np's at 37°C in PBS (pH 7.4). BDNF‐PLGA‐NPs (blue), GDNF‐PLGA‐NPs (red), and TGF‐ß3‐PLGA‐NPs (gray). Figure S4: When NPs were applied at various dosages to fibroblast cells and compared to control group cells, it was shown that NPs had no statistically significant effect on cell proliferation. As a consequence, it was shown that NPs did not affect cell proliferation and had no toxic impact. The proliferative or cytotoxic impact of the NPs on fibroblast cells was determined by WST‐1 analysis. Figure S5: Characterization of rat brain tissue‐derived NSCs at passage 3. Cell nuclei are labeled with DAPI (blue), GFAP, nestin, NG2, and S100 markers FITC (green) (Scale bar; 50 μm) (A). After 1 week of adherent culture, Nestin, Th, Tubb3, and Gfap gene expression levels of spontaneously differentiated NSCs were evaluated by RT‐PCR (B). Figure S6: Characterization of rat brain tissue‐derived pericytes at passage 3. Cell nuclei are labeled with DAPI (blue), positive for PDGFRβ (A) and NG2 (B, C) markers FITC (green), and α‐SMA (A, B) marker TR (red). Negative for GFAP TR (red) marker (Scale bar; 50 μm). [file CNS-31-e70576-s001.zip › CNS_70576_f1_FS1.tif]

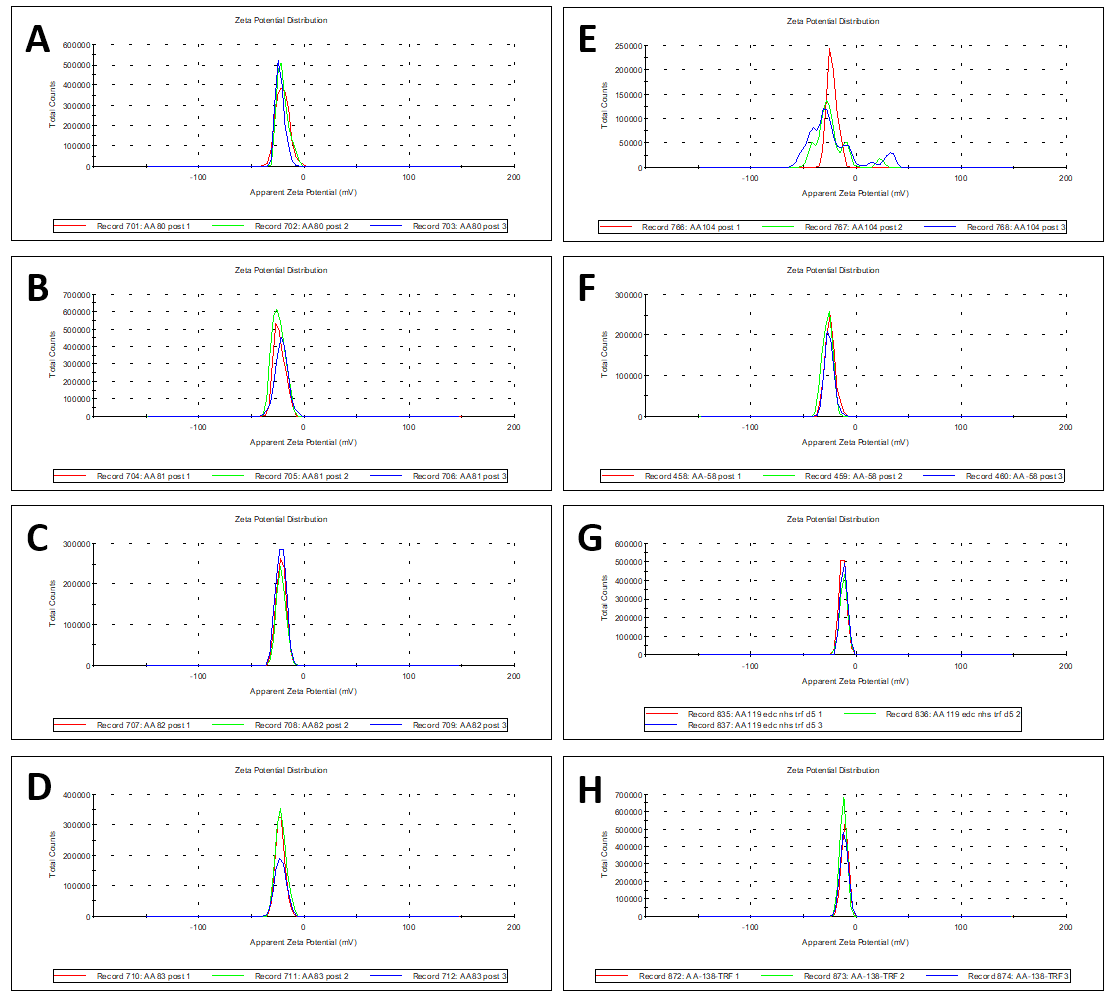

Supplement: Supplementary file 1 — Table S1: All differentiation experiment groups. Table S2: Primer sequences used in RT‐PCR. Table S3: NP uptake percentage findings from flow cytometry. Figure S1: Size distribution graphics of NPs (Malvern Zeta Sizer); PLGA‐NP (A), BDNF‐PLGA‐NP (B), GDNF‐PLGA‐NP (C), TGF‐ß3‐PLGA‐NP (D), C6‐PLGA‐NP (E), PLGA‐COOH‐NP (F), C6‐PLGA‐COOH‐NP (G), PLGA‐COOH‐NP‐TRF (H), and C6‐PLGA‐COOH‐NP‐TRF (I). Figure S2: Zeta potential graphics of NPs (Malvern Zeta Sizer); PLGA‐NP (A), BDNF‐PLGA‐NP (B), GDNF‐PLGA‐NP (C), TGF‐ß3‐PLGA‐NP (D), C6‐PLGA‐NP (E), PLGA‐COOH‐NP (F), PLGA‐COOH‐NP‐TRF (G), and C6‐PLGA‐COOH‐NP‐TRF (H). Figure S3: In vitro cumulative release profiles of growth factor‐loaded Np's at 37°C in PBS (pH 7.4). BDNF‐PLGA‐NPs (blue), GDNF‐PLGA‐NPs (red), and TGF‐ß3‐PLGA‐NPs (gray). Figure S4: When NPs were applied at various dosages to fibroblast cells and compared to control group cells, it was shown that NPs had no statistically significant effect on cell proliferation. As a consequence, it was shown that NPs did not affect cell proliferation and had no toxic impact. The proliferative or cytotoxic impact of the NPs on fibroblast cells was determined by WST‐1 analysis. Figure S5: Characterization of rat brain tissue‐derived NSCs at passage 3. Cell nuclei are labeled with DAPI (blue), GFAP, nestin, NG2, and S100 markers FITC (green) (Scale bar; 50 μm) (A). After 1 week of adherent culture, Nestin, Th, Tubb3, and Gfap gene expression levels of spontaneously differentiated NSCs were evaluated by RT‐PCR (B). Figure S6: Characterization of rat brain tissue‐derived pericytes at passage 3. Cell nuclei are labeled with DAPI (blue), positive for PDGFRβ (A) and NG2 (B, C) markers FITC (green), and α‐SMA (A, B) marker TR (red). Negative for GFAP TR (red) marker (Scale bar; 50 μm). [file CNS-31-e70576-s001.zip › CNS_70576_f2_FS2.tif]

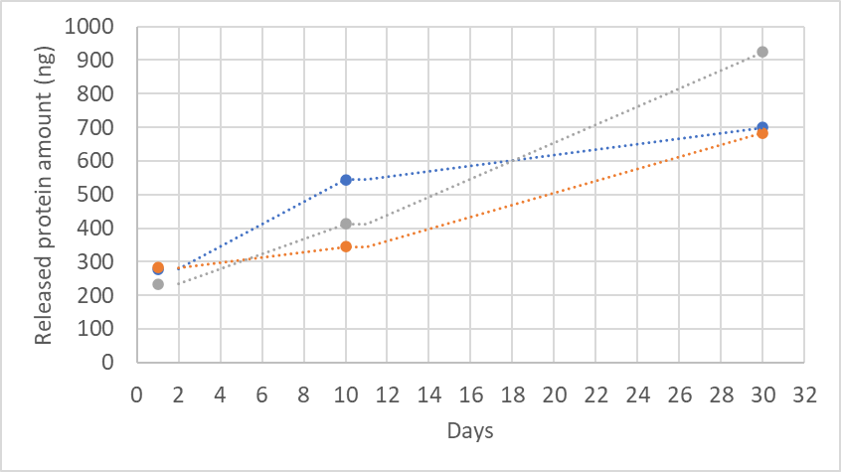

Supplement: Supplementary file 1 — Table S1: All differentiation experiment groups. Table S2: Primer sequences used in RT‐PCR. Table S3: NP uptake percentage findings from flow cytometry. Figure S1: Size distribution graphics of NPs (Malvern Zeta Sizer); PLGA‐NP (A), BDNF‐PLGA‐NP (B), GDNF‐PLGA‐NP (C), TGF‐ß3‐PLGA‐NP (D), C6‐PLGA‐NP (E), PLGA‐COOH‐NP (F), C6‐PLGA‐COOH‐NP (G), PLGA‐COOH‐NP‐TRF (H), and C6‐PLGA‐COOH‐NP‐TRF (I). Figure S2: Zeta potential graphics of NPs (Malvern Zeta Sizer); PLGA‐NP (A), BDNF‐PLGA‐NP (B), GDNF‐PLGA‐NP (C), TGF‐ß3‐PLGA‐NP (D), C6‐PLGA‐NP (E), PLGA‐COOH‐NP (F), PLGA‐COOH‐NP‐TRF (G), and C6‐PLGA‐COOH‐NP‐TRF (H). Figure S3: In vitro cumulative release profiles of growth factor‐loaded Np's at 37°C in PBS (pH 7.4). BDNF‐PLGA‐NPs (blue), GDNF‐PLGA‐NPs (red), and TGF‐ß3‐PLGA‐NPs (gray). Figure S4: When NPs were applied at various dosages to fibroblast cells and compared to control group cells, it was shown that NPs had no statistically significant effect on cell proliferation. As a consequence, it was shown that NPs did not affect cell proliferation and had no toxic impact. The proliferative or cytotoxic impact of the NPs on fibroblast cells was determined by WST‐1 analysis. Figure S5: Characterization of rat brain tissue‐derived NSCs at passage 3. Cell nuclei are labeled with DAPI (blue), GFAP, nestin, NG2, and S100 markers FITC (green) (Scale bar; 50 μm) (A). After 1 week of adherent culture, Nestin, Th, Tubb3, and Gfap gene expression levels of spontaneously differentiated NSCs were evaluated by RT‐PCR (B). Figure S6: Characterization of rat brain tissue‐derived pericytes at passage 3. Cell nuclei are labeled with DAPI (blue), positive for PDGFRβ (A) and NG2 (B, C) markers FITC (green), and α‐SMA (A, B) marker TR (red). Negative for GFAP TR (red) marker (Scale bar; 50 μm). [file CNS-31-e70576-s001.zip › CNS_70576_f3_FS3.tif]

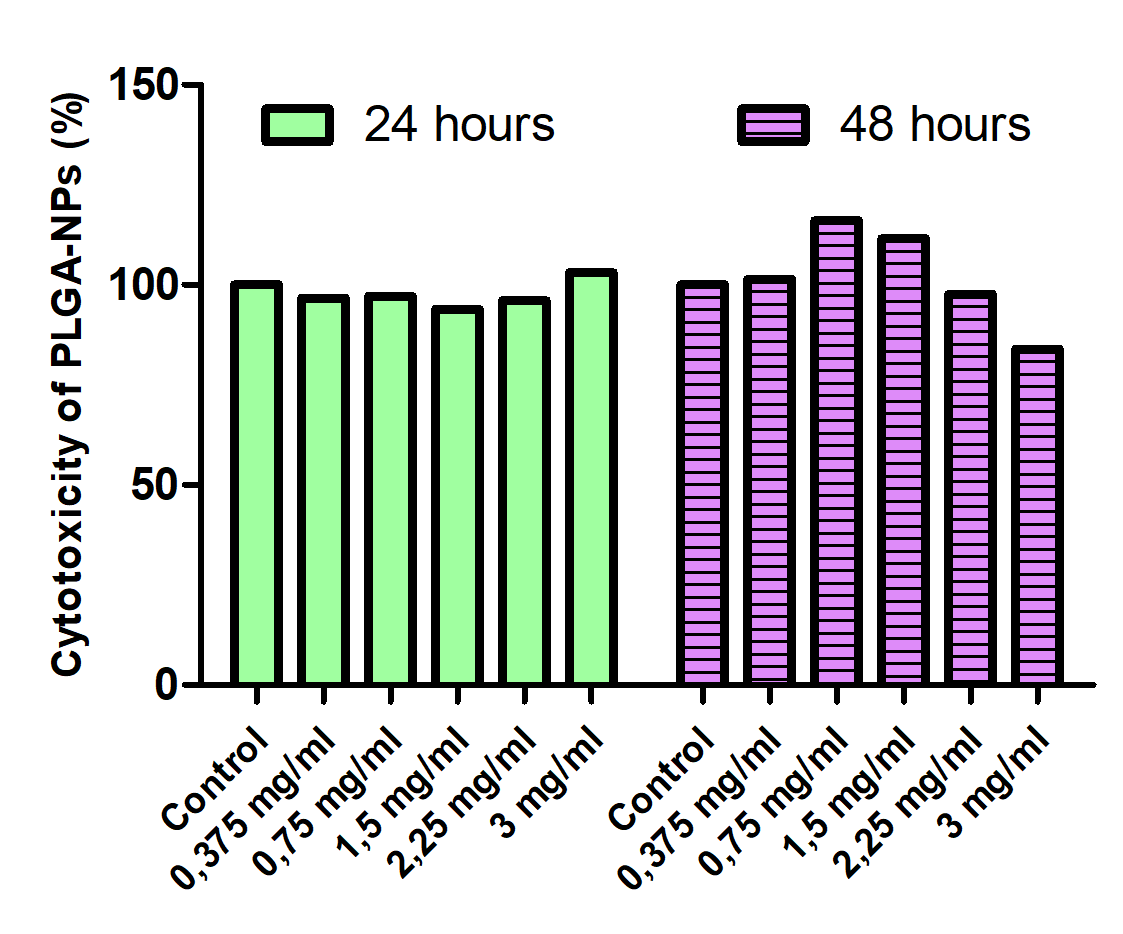

Supplement: Supplementary file 1 — Table S1: All differentiation experiment groups. Table S2: Primer sequences used in RT‐PCR. Table S3: NP uptake percentage findings from flow cytometry. Figure S1: Size distribution graphics of NPs (Malvern Zeta Sizer); PLGA‐NP (A), BDNF‐PLGA‐NP (B), GDNF‐PLGA‐NP (C), TGF‐ß3‐PLGA‐NP (D), C6‐PLGA‐NP (E), PLGA‐COOH‐NP (F), C6‐PLGA‐COOH‐NP (G), PLGA‐COOH‐NP‐TRF (H), and C6‐PLGA‐COOH‐NP‐TRF (I). Figure S2: Zeta potential graphics of NPs (Malvern Zeta Sizer); PLGA‐NP (A), BDNF‐PLGA‐NP (B), GDNF‐PLGA‐NP (C), TGF‐ß3‐PLGA‐NP (D), C6‐PLGA‐NP (E), PLGA‐COOH‐NP (F), PLGA‐COOH‐NP‐TRF (G), and C6‐PLGA‐COOH‐NP‐TRF (H). Figure S3: In vitro cumulative release profiles of growth factor‐loaded Np's at 37°C in PBS (pH 7.4). BDNF‐PLGA‐NPs (blue), GDNF‐PLGA‐NPs (red), and TGF‐ß3‐PLGA‐NPs (gray). Figure S4: When NPs were applied at various dosages to fibroblast cells and compared to control group cells, it was shown that NPs had no statistically significant effect on cell proliferation. As a consequence, it was shown that NPs did not affect cell proliferation and had no toxic impact. The proliferative or cytotoxic impact of the NPs on fibroblast cells was determined by WST‐1 analysis. Figure S5: Characterization of rat brain tissue‐derived NSCs at passage 3. Cell nuclei are labeled with DAPI (blue), GFAP, nestin, NG2, and S100 markers FITC (green) (Scale bar; 50 μm) (A). After 1 week of adherent culture, Nestin, Th, Tubb3, and Gfap gene expression levels of spontaneously differentiated NSCs were evaluated by RT‐PCR (B). Figure S6: Characterization of rat brain tissue‐derived pericytes at passage 3. Cell nuclei are labeled with DAPI (blue), positive for PDGFRβ (A) and NG2 (B, C) markers FITC (green), and α‐SMA (A, B) marker TR (red). Negative for GFAP TR (red) marker (Scale bar; 50 μm). [file CNS-31-e70576-s001.zip › CNS_70576_f4_FS4.tif]

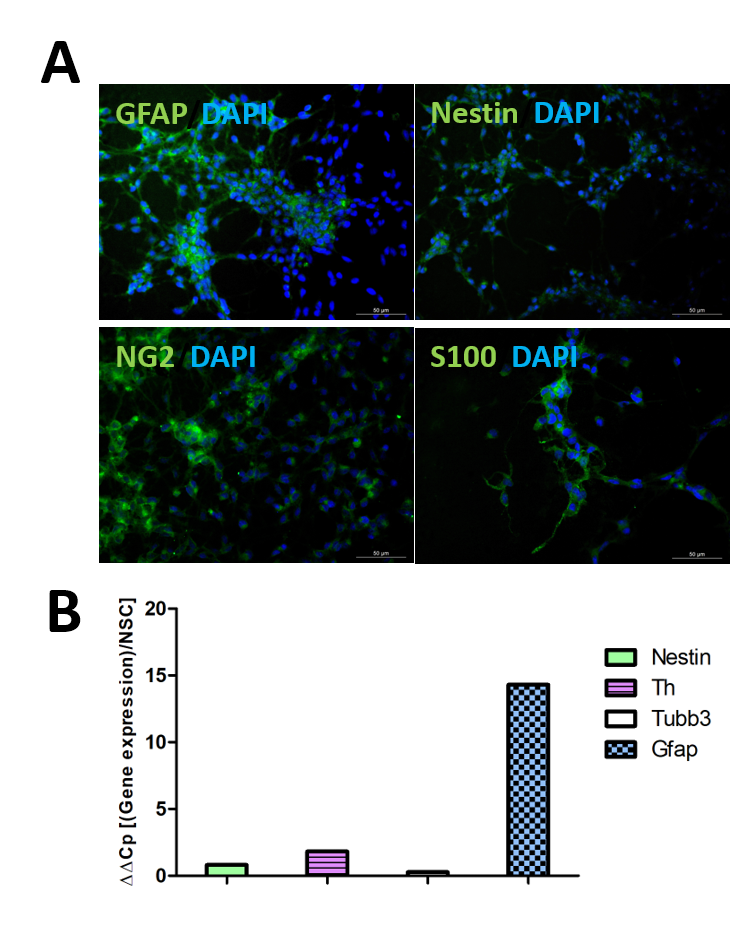

Supplement: Supplementary file 1 — Table S1: All differentiation experiment groups. Table S2: Primer sequences used in RT‐PCR. Table S3: NP uptake percentage findings from flow cytometry. Figure S1: Size distribution graphics of NPs (Malvern Zeta Sizer); PLGA‐NP (A), BDNF‐PLGA‐NP (B), GDNF‐PLGA‐NP (C), TGF‐ß3‐PLGA‐NP (D), C6‐PLGA‐NP (E), PLGA‐COOH‐NP (F), C6‐PLGA‐COOH‐NP (G), PLGA‐COOH‐NP‐TRF (H), and C6‐PLGA‐COOH‐NP‐TRF (I). Figure S2: Zeta potential graphics of NPs (Malvern Zeta Sizer); PLGA‐NP (A), BDNF‐PLGA‐NP (B), GDNF‐PLGA‐NP (C), TGF‐ß3‐PLGA‐NP (D), C6‐PLGA‐NP (E), PLGA‐COOH‐NP (F), PLGA‐COOH‐NP‐TRF (G), and C6‐PLGA‐COOH‐NP‐TRF (H). Figure S3: In vitro cumulative release profiles of growth factor‐loaded Np's at 37°C in PBS (pH 7.4). BDNF‐PLGA‐NPs (blue), GDNF‐PLGA‐NPs (red), and TGF‐ß3‐PLGA‐NPs (gray). Figure S4: When NPs were applied at various dosages to fibroblast cells and compared to control group cells, it was shown that NPs had no statistically significant effect on cell proliferation. As a consequence, it was shown that NPs did not affect cell proliferation and had no toxic impact. The proliferative or cytotoxic impact of the NPs on fibroblast cells was determined by WST‐1 analysis. Figure S5: Characterization of rat brain tissue‐derived NSCs at passage 3. Cell nuclei are labeled with DAPI (blue), GFAP, nestin, NG2, and S100 markers FITC (green) (Scale bar; 50 μm) (A). After 1 week of adherent culture, Nestin, Th, Tubb3, and Gfap gene expression levels of spontaneously differentiated NSCs were evaluated by RT‐PCR (B). Figure S6: Characterization of rat brain tissue‐derived pericytes at passage 3. Cell nuclei are labeled with DAPI (blue), positive for PDGFRβ (A) and NG2 (B, C) markers FITC (green), and α‐SMA (A, B) marker TR (red). Negative for GFAP TR (red) marker (Scale bar; 50 μm). [file CNS-31-e70576-s001.zip › CNS_70576_f5_FS5.tif]
